# Continuous infusion of piperacillin‐tazobactam significantly improves target attainment in children with cancer and fever

**DOI:** 10.1002/cnr2.1585

**Published:** 2021-11-18

**Authors:** Sabine F. Maarbjerg, Anders Thorsted, Lena E. Friberg, Elisabet I. Nielsen, Mikala Wang, Henrik Schrøder, Birgitte K. Albertsen

**Affiliations:** ^1^ Department of Pediatrics and Adolescent Medicine Aarhus University Hospital Aarhus Denmark; ^2^ Department of Pharmacy Uppsala University Uppsala Sweden; ^3^ Department of Clinical Microbiology Aarhus University Hospital Aarhus Denmark

**Keywords:** continuous infusion, dose optimization, febrile neutropenia, pharmacokinetics, piperacillin, target attainment

## Abstract

**Background:**

Children with febrile neutropenia commonly exhibit alterations of pharmacokinetic (PK) parameters, leading to decreased β‐lactam concentrations.

**Aims:**

This study evaluated piperacillin PK and probability of target attainment (PTA) with continuous infusion of piperacillin‐tazobactam, in order to optimize the dosing regimen.

**Methods:**

This prospective PK study included children with cancer, aged 1–17 years, who were treated with piperacillin‐tazobactam for suspected or verified infection. A piperacillin‐tazobactam loading dose (100 mg/kg) was administered followed by continuous infusion (300 mg/kg/day). The unbound fraction of piperacillin was quantified by high‐performance liquid chromatography and PK were described using population PK modeling. PK data was used to update and extend a previous PK model built on data following intermittent administration. Monte Carlo simulations were performed to assess PTA for targets of 100% time above the minimum inhibitory concentration (100% *f*T > MIC) and 50% *f*T > 4xMIC.

**Results:**

We included 68 fever episodes among 38 children with a median (IQR) age of 6.5 years and body weight of 27.4 kg (15.1–54.0). A three‐compartment model adequately described the concentration‐time data. Median (95% confidence interval) estimates for clearance and piperacillin concentration at steady state were 14.2 L/h/70 kg (13.0; 15.3) and 47.6 mg/L (17.2; 129.5), respectively. Body weight or lean body weight was significantly associated with the PK parameters, and body weight was integrated in the final PK model. Based on piperacillin exposure, continuous infusion was the only dosing regimen to achieve optimal PTA for the *P. aeruginosa* breakpoint (16 mg/L) with the target of 100% *f*T > MIC, and a daily dose of 300 mg/kg reached optimal PTA. The strict target of 50% *f*T > 4xMIC (64 mg/L) was not feasibly attained by any dosing regimen at recommended doses.

**Conclusion:**

Unlike conventional piperacillin intermittent administration and extended infusion regimens, continuous infusion allows the target of 100% *f*T > MIC to be reached for children with febrile neutropenia.

## 1 INTRODUCTION

Effective, empirical antimicrobial therapy improves therapeutic outcome and reduces infection‐related mortality in children with cancer and febrile neutropenia.^1^ Piperacillin‐tazobactam, a 𝛽‐lactam/𝛽‐lactamase‐inhibitor combination with broad‐spectrum activity, is frequently prescribed as empirical therapy.^1^, ^2^ Like other 𝛽‐lactams, piperacillin‐tazobactam exerts time‐dependent activity, in which bacterial killing is correlated to the percentage of time where the free drug concentration exceeds the pathogen's minimum inhibitory concentration (MIC) (*f*T > MIC). By maximizing *f*T > MIC, bactericidal and clinical effect is improved.^3^ Based on pre‐clinical data, maximum effect is achieved when the free drug concentration remains above MIC during 40–70% of the dosing interval.^4^, ^5^ Due to the lack of neutrophils in immunocompromised patients, stricter pharmacokinetic/pharmacodynamic (PK/PD) targets may be necessary for optimal antimicrobial efficacy.^3^, ^6^, ^7^

For less susceptible bacteria, conventional intermittent administration of piperacillin‐tazobactam is associated with a significant risk of not attaining the desired targets.^8^, ^9^, ^10^, ^11^ Pathophysiological disturbances, commonly observed in children undergoing aggressive chemotherapy, can widely affect the pharmacokinetics (PK) of antimicrobials, and alterations in volume of distribution and clearance reduce the likelihood of adequate pharmacodynamic (PD) coverage.^10^, ^12^, ^13^ Prolongation of the infusion time, through extended infusion or continuous infusion, form an attractive strategy to maximize *f*T > MIC without increasing the daily dose. Although a large randomized trial^14^ found no outcome difference between intermittent administration and continuous infusion of β‐lactams in adults, prolonged infusions have demonstrated improved probability of target attainment (PTA) and clinical outcome, including mortality.^15^, ^16^, ^17^ Published pediatric studies advocate a PTA benefit with extended infusion and, in particular, continuous infusion of piperacillin‐tazobactam. However, available data is mainly based on simulations from PK models built on intermittent administration or extended infusion regimens in small, specific populations.^8^, ^9^, ^10^, ^11^, ^18^, ^19^, ^20^, ^21^, ^22^, ^23^ Only a single, small study^7^ has prospectively evaluated and demonstrated increased PTA with continuous infusion in neutropenic children.

The aim of this study was to evaluate piperacillin PK and PTA in children with cancer receiving continuous infusion of piperacillin‐tazobactam, in order to define an optimal dosing regimen and substantiate the use of continuous infusion in a clinical setting.

## 2 METHODS

### 2.1 Study design and patient population

This prospective, single‐center PK study was conducted at the Department of Pediatrics and Adolescent Medicine, Aarhus University Hospital, Denmark between 1 August 2018 and 30 June 2020. The study was approved by the Central Denmark Region Committee on Health Research Ethics (registration no. 1‐10‐72‐427‐17), the Danish Medicines Agency (Clinicaltrialsregister.eu registration no. 2017‐004281‐10) and the Danish Data Protection Agency (registration no. 1‐16‐02‐924‐17). Written informed consent was obtained from both parents or legal guardians. In accordance with Danish law, children aged 15–17 years were allowed to give written informed consent for themselves, in close collaboration with their parents or legal guardians.

Children, aged 1–17 years, with cancer who received empirical piperacillin‐tazobactam to treat a suspected or documented infection were eligible for inclusion. Exclusion criteria covered; fully breastfeed infants, history of anaphylaxis to 𝛽‐lactams, admission to intensive care unit and body weight <8 kg. Eligible children could be enrolled several times in case of recurrent fever episodes. Fever was defined as a single temperature measurement above 38.5°C and a recurrent fever episode as fever after seven fever‐free days. Clinical variables and baseline demographics were registered: sex, age, body weight, height, cancer diagnosis, fever duration, piperacillin‐tazobactam bolus dosing prior to initiation of continuous infusion, plasma creatinine, C‐reactive protein, absolute neutrophil count and microbiological isolates detected in blood cultures.

### 2.2 Drug dosing and blood sample collection

As standard care, piperacillin‐tazobactam was prescribed at 300 mg/kg/day and administered intermittently every 8 h (qh8). After study enrollment, eligible patients received a 2–5 min piperacillin‐tazobactam loading dose of 100 mg/kg (not subtracted from the total daily dose) followed by continuous infusion of 300 mg/kg/day (piperacillin component, maximum of 16 000 mg/day) at a fixed rate over 24 h. A supplemental loading dose of 100 mg/kg (maximum four doses per day) was administered in case of a discontinued infusion for longer than 30 min.

The blood sampling schedule was designed, using optimal design theory, in an attempt to accurately inform the piperacillin concentration‐time course with continuous infusion, and each patient was scheduled to contribute three blood samples. Sampling times covered peak concentration immediately after loading dose and prior to continuous infusion initiation (2–30 min), distribution phase (0.5–1.5 h), and steady state concentration (12–24 h). Most study participants had indwelling catheters with two separate infusion lines, and piperacillin samples were drawn from the infusion line that was not occupied by the continuous infusion. In case of a single line‐catheter, the samples were obtained after thorough saline flushing of the catheter.

### 2.3 Ultra‐high performance liquid chromatography

The unbound piperacillin concentrations in serum were quantified using validated ultra‐high performance liquid chromatography (HPLC) following ultra‐filtration (Agilent 1290, Agilent Technologies, USA), as formerly described.^24^, ^25^ Intra‐run (total) imprecisions (coefficients of variation [%]) were 10.2% (15.3%) at 4.5 mg/L and 4.7% (8.2%) at 15.6 mg/L. The limit of quantification for piperacillin was 0.5 mg/L, and was defined as the lowest concentration with a coefficient of variation <20%. The tazobactam component of piperacillin‐tazobactam was not quantified.

### 2.4 PK/PD targets and MIC profile

PTA was assessed for PK/PD targets of 100% *f*T > MIC (free piperacillin concentration sustained above MIC throughout the dosing interval) and 50% *f*T > 4xMIC. Due to the steady state concentration‐time profile arising from continuous infusion, 50% *f*T > 4xMIC and 100% *f*T > 4xMIC result in similar PTA. The PK/PD targets were evaluated in relation to the susceptibility breakpoint MIC for *Pseudomonas aeruginosa* (16 mg/L), published by the European Committee on Antimicrobial Susceptibility Testing (EUCAST),^26^ and MIC_50_ (2 mg/L) and MIC_90_ (4 mg/L), calculated from an institutional MIC distribution of bacteria in blood cultures from pediatric cancer patients through 10 years.^27^

### 2.5 Pharmacokinetic modeling

Piperacillin samples obtained during continuous infusion were merged with samples previously obtained during intermittent administration in the same patient population^9^, ^20^ (482 samples from 43 patients across 89 fever episodes). An extended population PK model was build based on the merged data. The two‐compartment model,^9^, ^20^ with linear elimination and allometric (fixed exponents) scaling of PK parameters to body weight, was used as starting point. The model structure (two or three compartments) and inter‐individual as well as inter‐occasion (fever episode) variability were re‐evaluated. Previously insignificant structural components, namely kidney maturation^28^ and non‐linear clearance, were reassessed and parameter‐covariate relations were tested by stepwise covariate modeling: sex, age, underlying malignancy, bacteraemia, neutropenia, glomerular filtration rate (GFR, estimated by Schwartz equation),^29^ fever duration and peak temperature. A bootstrap analysis with 2000 samples was performed for the final model to obtain 95% confidence intervals for the model parameters as a measure of uncertainty.

PK modeling was performed using NONMEM version 7.4.4 (ICON Development Solutions, Gaithersburg, MD)^30^ facilitated by Perl‐Speaks‐NONMEM.^31^ Statistical selection of a suitable model structure was performed by the likelihood‐ratio test of the objective function values (OFV). Model selection and evaluation was guided by residual goodness‐of‐fit plots and visual predictive checks of simulated concentration‐time profiles. Descriptive statistics were performed using STATA 15.1 software (STATA Corp, College Station, TX), and *p* < .05 were considered statistically significant.

### 2.6 Monte Carlo simulations

Based on the updated PK model and individual PK parameter estimates, concentration‐time profiles and PTA were predicted for each individual fever episode. Monte Carlo simulations were performed to assess the proportion of children attaining the PK/PD targets with alternative dosing regimens. The simulated dosing regimens covered intermittent administration every six (q6h) and eight hours (q8h), extended infusion (infusion for half of the dosing interval, administered q6h and q8h) and continuous infusion. Daily doses of 300 and 400 mg/kg (maximum 16 000 mg daily) were simulated for all dosing regimens. A population of 10 000 children aged 2–18 years with equal sex distribution was constructed based on the NHANES database (1999–2015)^32^ with median (2.5th–97.5th percentiles) body weight of 42.7 kg (12.6–94.2 kg). PTA was predicted over MICs from 0.125 to 128 mg/L for body weight groups of <25 kg, ≥25 to <50 kg, ≥50 to <75 kg, and ≥75 kg. To assess the likelihood of treatment success in this population, the cumulative fraction of response (CFR)^21^, ^33^ was estimated for each regimen; PTA and the proportion of isolates at each MIC within the institutional MIC distribution^27^ were multiplied and summed. A dosing regimen was considered successful if PTA or CFR ≥95%.

## 3 RESULTS

### 3.1 Study population

A total of 38 children with cancer received continuous infusion of piperacillin‐tazobactam across 68 fever episodes (1–4 per child). Median (IQR) for age, body weight and GFR were 6.5 years (4; 15), 27.4 kg (15.1; 54.0) and 175.5 ml/min/1.73 m^2^ (133.3; 209.3), respectively, and a bacteraemia was detected in six of 68 (9%) fever episodes. This cohort was merged with the intermittent administration cohort that comprised 43 children and 89 fever episodes,^9^, ^20^ resulting in PK data from 78 children (three children were enrolled in both cohorts) across 157 fever episodes. Clinical and demographic characteristics of the continuous infusion and intermittent administration cohorts are summarized in Table 1. From a clinical point of view, median age (IQR) differed between the two cohorts (6.5 [4; 15] vs. 12 [7; 14] years), however, the difference was statistically insignificant. The only characteristic that differed significantly was the proportion of fever episodes caused by “local infections” (*p* = .033).

**TABLE 1:** Clinical characteristics of children and fever episodes in CI cohort, IA cohort and total cohort (CI + IA)

Characteristic	CI cohort	IA cohort	Total population (CI + IA)^a^	*p*
Included patients	*n* = 38	*n* = 43	*n* = 78	
Gender, *n* (%)				
Male	24 (63%)	27 (63%)	49 (63%)	0.995
Female	14 (37%)	16 (37%)	29 (37%)	
Age (years)	6.5 (4–15)^b^	12 (7–14)	10.5 (5–14)	0.174
Body weight (kg)	27.4 (15.1–54.0)	39.4 (22.5–50.4)	31.6 (20–51)	0.141
Body surface area (m^2^)	0.97 (0.64–1.60)	1.31 (0.90–1.58)	1.12 (0.77–1.58)	0.109
Underlying malignancy, *n* (%)				
Hematological malignancies	17 (45%)^c^	18 (42%)	32 (42%)	0.868
Solid tumors	21 (55%)^d^	25 (58%)	46 (59%)	
GFR (ml/min/1.73 m^2^)^5^	175.5 (133.3–209.3)	171.5 (147.8–208.4)	176.1 (136.6–209.9)	0.923
Fever episodes	*n* = 68	*n* = 89	*n* = 157	
Positive blood culture, *n* (%)	6 (9%)	10 (11%)	16 (10%)	1.000
*Viridans streptococci*		3 (30%)	3 (19%)	
*Gram‐positive cocci, unspecified*	1 (17%)		1 (6%)	
*Pseudomonas aeruginosa*	1 (17%)		1 (6%)	
*Micrococcus*	2 (33%)	3 (30%)	5 (31%)	
*CoNS*	2 (33%)	2 (20%)	4 (25%)	
*Staphylococcus aureus*		1 (10%)	1 (6%)	
*Klebsiella pneumoniae*		1 (10%)	1 (6%)	
Fever of unknown origin	44 (65%)	69 (78%)	113 (72%)	0.100
Local infection	18 (26%)	10 (11%)	28 (18%)	0.033
GFR (ml/min/1.73 m^2^)^e^	174.9 (150.4;225.7)	172.4 (139.8–210.8)	174.4 (144.1–220.8)	0.916
Neutropenia (x10^9^/L)^f^	49 (72%)	77 (87%)	126 (80%)	0.126
Duration of fever (days)	3.5 (3–4)	1 (1–3)	3 (3–4)	0.245
CRP (mg/L)	34.5 (19.7–54.9)	‐	‐	
Maximum CRP (mg/L)	80.9 (44.5–135.9)	‐	‐	
No. of piperacillin‐tazobactam bolus doses before start of CI^g^	5.5 (3–9)	‐	‐	
CI disruption >30 min	9/68 (13%)			

*Note*: Continuous variables are presented as medians (IQR) and dichotomous data are presented as a number (%).

Abbreviations: ANC, Absolute Neutrophil Count; CI, Continuous infusion; CoNS; Coagulase negative staphylococci; CRP, C‐reactive protein; IA, Intermittent administration; IQR, Interquartile range.

a Three children were enrolled in both IA and CI cohort, thus *n* = 78 (total population).

^b^ Age distribution: 0–5 years: *n* = 16; >5–10 years: *n* = 5; >10–15 years; *n* = 8; >15 years: *n* = 9.

^c^ Hematological malignancies; acute lymphoblastic leukemia (*n* = 9), acute myeloid leukemia (*n* = 3), unspecified leukemia (*n* = 2), B‐cell Non‐Hodgkin lymphoma (*n* = 1), Hodgkins lymphoma (*n* = 1), myelomatosis (*n* = 1).

^d^ Solid tumors; medulloblastoma (*n* = 4), neuroblastoma (*n* = 3), osteosarcoma (*n* = 2), langerhans histiocytosis (*n* = 1), other sarcomas (*n* = 1), Ewing sarcoma (*n* = 9), Wilms tumor (*n* = 1).

^e^ GFR: glomerular filtration rate calculated from the Schwartz equation^28^; GFR = kL/Pcr (L: body length in cm, Pcr: plasma creatinine concentration in mg/dl and k: constant of proportionality).

^f^ Neutropenia: ANC < 0.5 x 10^9^/L on the day of admission.

^g^ Range (1–39).

### 3.2 Pharmacokinetic modeling and covariates

A total of 192 plasma piperacillin samples were collected during continuous infusion, with a median number (range) of samples per study subject of two (two‐three), and the extended PK model was built on 674 piperacillin samples in total. Data were best described by a three‐compartment model with a fast distribution phase, which improved the model fit significantly (ΔOFV = 118; Figure S1), and no other structural model improvements were identified. As continuous infusion samples, particularly those at steady state, showed high variability, model fit was significantly improved by adding a separate residual error (residual unexplained variability) for the continuous infusion data (ΔOFV = 141; 65 vs. 27.5% for intermittent administration data). However, residual unexplained variability was reduced to 54% by allowing a separate inter‐occasion variability in clearance in the continuous infusion cohort (ΔOFV = 17.4) and inter‐individual variability in the residual error (ΔOFV = 16.0), thereby allowing higher residual error in samples from some individuals. For covariates, body weight was significantly associated with the PK parameters, that is, clearance, inter‐compartmental clearances, and volumes of distribution. Incorporation of lean body weight rather than total body weight further improved model fit (ΔOFV = 20.8). Still, total body weight was retained as a covariate in the final model, since parameter and variability estimates remained unchanged, and as pediatric dosing typically rely on total body weight. No other statistically significant covariate‐parameter associations were identified. Simulation‐based, visual predictive checks and residual diagnostics showed adequate description of the concentration‐time course during continuous infusion without systematic bias (Figure S1). Compared with the previous model, only minor changes in the PK parameter estimates were identified, and the final PK parameters are summarized in Table 2.

**TABLE 2:** Pharmacokinetic parameters

Parameter	Parameter description	Estimate	95%CI^a^
CL (L/h)	Elimination clearance	14.24^b^	(12.98, 15.27)
Vc (L)	Central volume of distribution	5.953^b^	(3.468, 7.467)
Q_1_ (L/h)	Inter‐compartmental clearance (slow)	0.1943^b^	(0.1548, 0.2322)
Vp_1_ (L)	Peripheral volume of distribution (slow)	3.537^b^	(1.968, 5.277)
Q_2_ (L/h)	Inter‐compartmental clearance (rapid)	27.45^b^	(19.58, 37.71)
Vp_2_ (L)	Peripheral volume of distribution (rapid)	7.329^b^	(6.140, 8.627)
CL IOV_IA_ (%)	Inter‐individual variability in CL (IA cohort)	18.0	(14.0, 21.8)
CL IOV_CI_ (%)	Inter‐individual variability in CL (CI cohort)	48.1	(30.0, 67.9)
ERR IIV (%)	Inter‐individual variability in residual error	30.7	(19.0, 43.3)
ERR_IA_ (%)	Residual unexplained variability (IA cohort)	27.5	(23.9, 30.8)
ERR_CI_ (%)	Residual unexplained variability (CI cohort)	50.0	(38.4, 61.1)

^a^ Based on a non‐parametric bootstrap of the data set (with 1837/2000 successful samples).

^b^ Estimates for 70 kg, scaled to individual body weight according to: CL_typical,individual_ = CL_typical,70kg_·(WT_individual,kg_/70_kg_)^0.75^ and *V*_typical,individual_ = *V*_typical,70kg_·(WT_individual,kg_/70_kg_)^1.0^.

**FIGURE 1:**
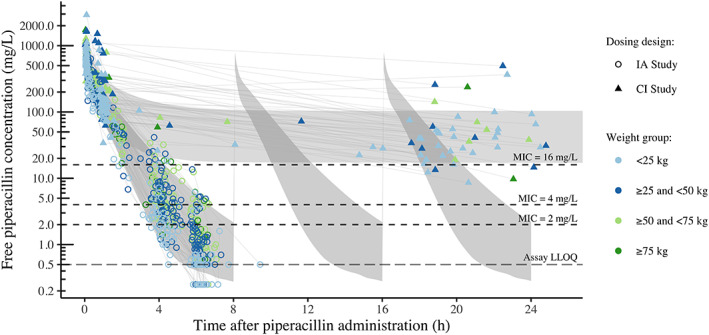
Piperacillin concentration‐time course for intermittent administration every 8 h (circles) and continuous infusion (triangles) of piperacillin‐tazobactam (both 300 mg/kg/day), with the body weights indicated (point color). The gray shaded areas represent the 95% prediction interval of individual predictions based on the final model. CI, continuous infusion; IA, intermittent administration; MIC, minimum inhibitory concentration

Figure 1 illustrates the different piperacillin concentration‐time course with intermittent administration and continuous infusion with 95% prediction interval of individual predictions. A sample was available at steady state in 49 of 68 (72%) fever episodes, and observed median piperacillin concentration (95% confidence interval) was of 47.6 mg/L (17.2; 129.5). The observed steady state concentrations showed high variability, and were spread above and below the model‐predicted concentrations, with a few observations placed outside the 95%‐prediction interval. Based on observed piperacillin exposure, 89.9 and 22.4% of the children achieved the targets of 100% *f*T > MIC and 50% (100% for continuous infusion) *f*T > 4xMIC for the *P. aeruginosa* breakpoint, respectively.

### 3.3 Probability of target attainment

**FIGURE 2:**
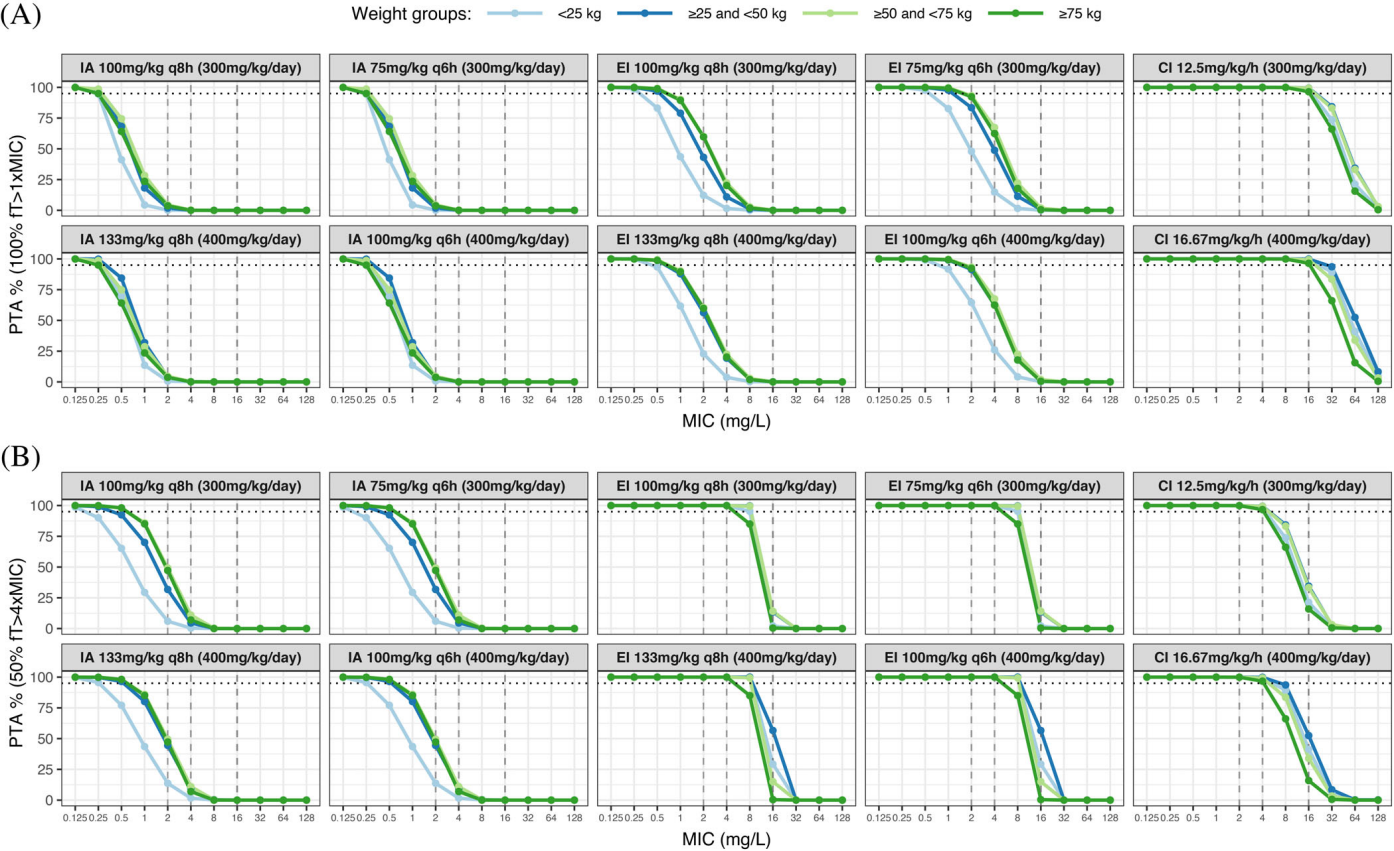
Probability of target attainment for the two targets (A) 100% *f*T > MIC and (B) 50% *f*T > 4xMIC (effectively 100% *f*T > 4xMIC for continuous infusion). PTA was simulated for intermittent administration every eight (q8h) and 6 h (q6h), extended infusion q8h and q6h and lasting half of a dosing interval, and continuous infusion. The colors represent different weight groups and MIC_50_ (2 mg/L), MIC_90_ (4 mg/L), and the EUCAST MIC breakpoint for *P. aeruginosa* (16 mg/L) are represented by the dashed vertical lines. The dashed horizontal lines illustrate that 95% of the simulated population have reached the specified *f*T > MIC target. CI, continuous infusion; EI, extended infusion; IA, intermittent administration; MIC, minimum inhibitory concentration; PTA, probability of target attainment

**FIGURE 3:**
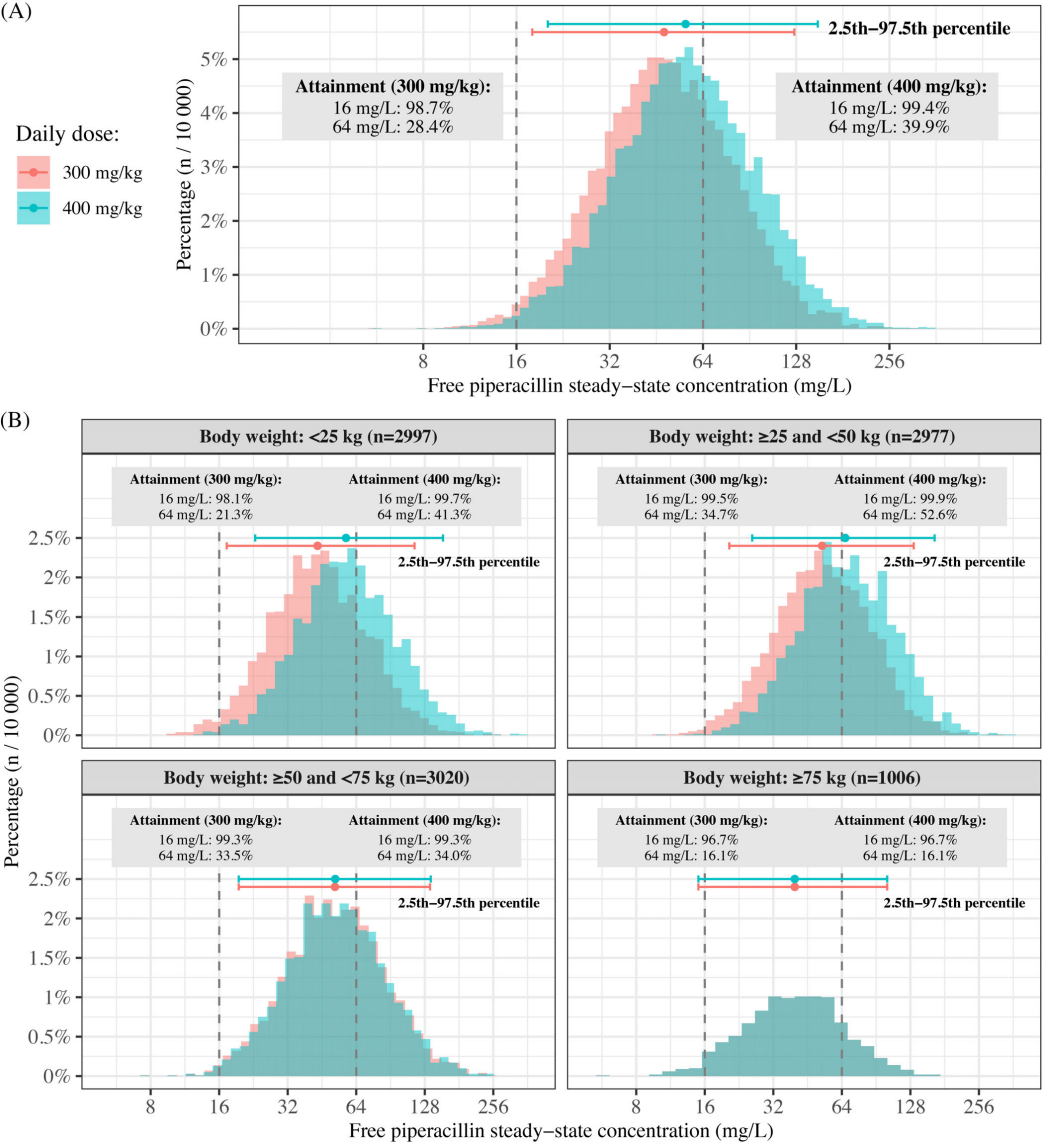
Steady state piperacillin concentrations for continuous infusion of 300 and 400 mg/kg/day (maximum 16 000 mg/day) according to body weight in 10 000 simulated children. (A) Concentrations for the complete population, with 95th interval (2.5th to 97.5th percentile) indicated to enable comparison with the *P. aeruginosa* breakpoint for the two targets of 100% *f*T > MIC (16 mg/L) and 50/100% *f*T > 4xMIC (64 mg/L). (B) The 10 000 children divided into body weight categories, with the distribution and 95th interval indicated. The maximum daily dose is reached at 53.3 kg (300 mg/kg) and 40 kg (400 mg/kg), resulting in overlapping distributions. CI, continuous infusion; MIC, minimum inhibitory concentration

PTA for the two PK/PD targets is illustrated in Figure 2 and Table 3. Predictions of median steady state concentrations (95% percentiles) for continuous infusion of 300 mg/kg/day was 47.9 mg/L (18.0; 126) and 56.2 mg/L (20.2; 150) for 400 mg/kg/day (Figure 3A). For the *P. aeruginosa* breakpoint (16 mg/L), simulations based on the updated PK model confirmed the findings of the previous model‐predictions showing that continuous infusion was required to reach the target of 100% *f*T > MIC (Figure 2A). With continuous infusion at doses of 300 mg/kg/day and 400 mg/kg/day, 98.7 and 99.4% of the children achieved this target, respectively. Intermittent administration and extended infusion regimens achieved MICs of 0.25 and 2 mg/L (MIC_50_) with the target of 100% *f*T > MIC, respectively.

**TABLE 3:** Probability of target attainment and cumulative fraction of response for various dosing regimens for PK/PD targets of 100% *f*T > MIC and. 50% *f*T > 4xMIC (100% *f*T > 4xMIC for continuous infusion)

Dosing regimen		PTA OF MIC_50_ (2.0 mg/L)		PTA OF MIC_90_ (4.0 mg/L)		PTA OF *P.A*. breakpoint (16.0 mg/L)		CFR (%)	
		100% *f*T > MIC	50% *f*T > 4xMIC	100% *f*T > MIC	50% *f*T > 4xMIC	100% *f*T > MIC	50% *f*T > 4xMIC	100% *f*T > MIC	50% *f*T > 4xMIC
IA 300 mg/kg/day	q8h	2.30%	30.90%	0.10%	5.50%	0%	0%	2.00%	32%
	q6h	17.90%	67.90%	3.10%	27.10%	0%	0%	15.70%	60.90%
IA 400 mg/kg/day	q8h	3.10%	37.00%	0.20%	7.40%	0%	0%	2.70%	31.80%
	q6h	22.00%	76.60%	3.80%	35.00%	0%	0%	19.30%	69.30%
EI 300 mg/kg/day	q8h	40.60%	100%	12.50%	100%	0%	8.90%	40.10%	96.00%
	q6h	76.70%	100%	45.60%	100%	0.90%	8.90%	70.30%	96.00%
EI 400 mg/kg/day	q8h	47.90%	100%	15.70%	100%	0%	30.20%	42.70%	96.60%
	q6h	84.00%	100%	53.10%	100%	1.20%	30.20%	78.50%	96.60%
CI 300 mg/kg/day		100%	100%	100%	98.70%	98.70%	28.30%	98.70%	96.30%
CI 400 mg/kg/day		100%	100%	100%	99.40%	99.40%	39.80%	98.70%	96.80%

*Note*: IA, intermittent administration; EI, Extended infusion; CI, continuous infusion; % *f*T > MIC, Percentage of the dosing interval with a free piperacillin concentration above MIC; PTA, probability of target attainment; P.A breakpoint, *Pseudomonas aeruginosa* breakpoint; CFR, Cumulative fraction of response. q6h + q8h: administration every 6 or 8 h.

For the target of 50%/100% *f*T > 4xMIC, none of the tested dosing regimens reached the *P. aeruginosa* breakpoint (64 mg/L; Figure 2B). Simulations from the updated model showed higher PTA for continuous infusion of 300 and 400 mg/kg/day; in which 28.4 and 39.9% of children achieved this target, respectively (Figure 3), and 30.2% with extended infusion of 400 mg/kg/day q6h. Similar to the previous model, intermittent administration showed insufficient PTA with this target. To achieve a MIC of 64 mg/L in 95% of the patients, continuous infusion of potentially toxic doses of 705–1018 mg/kg/day were required, dependent on body weight (Figure S2). PTA with continuous infusion was not significantly affected by body weight.

## 4 DISCUSSION

Children with febrile neutropenia commonly exhibit physiological alterations, which lead to decreased piperacillin concentrations. Selection of an optimal empirical dosing regimen is challenged by limited pharmacokinetic data. Simulations from our previous PK model,^9^, ^20^ built on intermittent administration data, suggested that continuous infusions achieve optimal PTA for the *P. aeruginosa* breakpoint. In order to validate these findings in a clinical study and define optimal dosing regimens, piperacillin PK and PTA were evaluated in a prospective study in children with cancer receiving continuous infusion, and the PK data was used to extend the previous PK model.

Results from the updated PK model confirmed the findings of the previous model‐predictions. Unlike intermittent administration and extended infusion regimens, continuous infusion reached optimal PTA for a MIC of 16 mg/L with the 100% *f*T > MIC target, and daily doses of 300 and 400 mg/kg achieved nearly equivalent PTA (98.7 vs. 99.4%; Figure 2 and Table 3). None of the tested dosing regimens reached the strict target of 50/100% *f*T > 4xMIC (64 mg/L). The higher PTA with extended infusion (400 mg/kg/day) and continuous infusion regimens (approximately 30 vs. 18%), identified in the updated model, may be explained by the slightly lower estimate of clearance in the updated model (15.4 and 14.2 L/h), the key parameter governing steady‐state concentrations achieved with prolonged infusions.

To date, only one relatively small trial^7^ (19 patients) has prospectively evaluated and demonstrated increased PTA with continuous infusion in neutropenic children. Contrary to our findings, Delvallée et al found that continuous infusion of 400 mg/kg/day was necessary to cover the *P. aeruginosa* breakpoint, which may be explained by the very strict target (100% *f*T > 6xMIC), evaluated in this study. Apart from the Delvallée study, available data in neutropenic children is limited to Monte Carlo simulations derived from PK models of β‐lactams administered intermittently.^8^, ^9^, ^34^ Consistent with our findings, simulations in neutropenic children^8^, ^9^, ^34^ demonstrated poor PTA with intermittent administration and PTA improvement with prolonged infusions at recommended doses. Similar results have been reported in non‐neutropenic children^11^, ^18^, ^19^, ^21^, ^22^ and in neutropenic adults.^35^, ^36^, ^37^

The PK parameters in the updated model differed only slightly from the previous model estimates,^9^, ^20^ presumably due the comparable patient populations (Table 1), and the minor changes in parameter estimates support the validity of the previous model. The estimated clearances in the two PK models (15.4 and 14.2 L/h) were comparable to the high clearances previously reported among severely ill children,^8^, ^10^, ^11^, ^18^, ^22^ (ranging from 12.6 to 20.9 L/h), and likewise, for the estimated central volume of distribution in our models (16.0 and 13.3 L) compared with previous studies (range 9.0–30.1 L).^8^, ^10^, ^11^, ^18^, ^22^ Expansion to a three‐compartment model was driven by samples collected shortly after loading dose, with limited impact on the terminal profile that drives PTA. Increased residual variability and higher variability in piperacillin clearance between fever episodes were detected in individuals who received continuous infusion (Figure 1). This may partly result from the steady state profile following continuous infusion being more sensitive to fluid shifts or psychical activity, and the drug concentrations may be directly influenced by small variations in infusion rate or breaks.

Piperacillin is mainly cleared renally through glomerular filtration and saturable tubular secretion. Both processes complete maturation by the age of 1–2 years, however, renal size and capacity continue to increase during childhood.^12^ No significant improvements were observed when incorporating kidney maturation into the PK model, likely due to a limited number of very young children in the study. Concentrations arising from continuous infusion were around the *K*_m_'s (Michaelis Menten constant) previously reported,^38^, ^39^ however, the combined analysis of continuous infusion and intermittent administration data did not reveal a non‐linear clearance mechanism. This may result from quantification of unbound, rather than total, piperacillin concentrations, assuming that non‐linearities are present in protein binding.

During continuous infusion, median concentrations at steady state were considerably higher than following comparable doses administered intermittently.^9^, ^20^ Although piperacillin has a wide safety margin, the risk of adverse events, particularly neurotoxicity, increases with higher drug concentrations achieved by high‐dose intermittent administration or continuous infusion. While no clear cut off threshold for neurotoxicity has been established, a threshold of 157.2 mg/L was proposed in adults,^40^ suggesting that very high concentrations may be tolerated without severe adverse events. In children with cystic fibrosis, piperacillin doses of 600 mg/kg daily proved safe,^41^ however, safety data on high doses in neutropenic children are lacking. Concentrations above the therapeutic interval (defined as 100% *f*T > 2‐4 x *P. aeruginosa* breakpoint MIC; 33–64 mg/L), and particularly >100 mg/L, were recently associated with significantly increased mortality.^42^ It was not reported whether subtherapeutic concentrations were associated with a poorer outcome. Achievement of the *P. aeruginosa* breakpoint with 4xMIC requires piperacillin concentrations (64 mg/L) significantly below the suggested neurotoxicity threshold and likewise, the maximum steady state concentrations of 56.2 mg/L, attained by continuous infusion of 400 mg/kg/day in this study, remained significantly below this threshold.

To avoid high and potentially harmful piperacillin doses with intermittent administration, prolonged infusions at recommended doses are a safe approach to improve *f*T > MIC. The PTA benefit with prolonged infusions becomes increasingly evident in bacteria with elevated MICs. Although bloodstream infections caused by *P. aeruginosa* are relatively rare among children with cancer, they represent a worst‐case scenario in terms of susceptibility and are often associated with a severe clinical course. In this study, *P. aeruginosa* accounted for one of six (17%) blood culture isolates and for 15 of 457 (3%) within the institutional MIC distribution^27^; all isolates were susceptible to piperacillin‐tazobactam (maximum MIC of 4 mg/L). Recent guidelines^2^ for the treatment of pediatric febrile neutropenia recommend anti‐pseudomonal coverage, and our findings confirmed that continuous infusion was the optimal dosing regimen to achieve the *P. aeruginosa* breakpoint with the 100% *f*T > MIC target. A daily dose of 300 mg/kg (preceded by a 100 mg/kg loading dose) provided optimal PTA, and raising the daily dose to 400 mg/kg would not noticeably increase PTA (Table 3). Due to large PK variability in critically ill patients, therapeutic drug monitoring could be considered in high‐risk pediatric febrile neutropenia to maximize piperacillin exposure against the least susceptible pathogens.

Extrapolations of optimal PK/PD targets from preclinical studies, where plasma PK are simulated, are complicated by the variable tissue penetration, frequently observed in severely ill patients.^33^ While consensual PK/PD targets have not yet been established in children with febrile neutropenia, While there is growing evidence supporting the use of more stringent targets of 50–100% *f*T > 4‐6xMIC in severely ill and neutropenic patients,^3^, ^8^, ^33^, ^43^, ^44^ to compensate for the reduced immunological response and limited post‐antibiotic effect.^3^, ^8^ This explains the rationale for evaluating relatively strict PK/PD targets in this study. However, none of the evaluated dosing regimens achieved optimal PTA for the strict target of 50/100% *f*T > 4xMIC in this study.

Continuous infusion of potentially harmful doses up to 1018 mg/kg/day were required to reach the target of 50/100% *f*T > 4xMIC (Figure S2), which proves that very strict targets are not feasibly attained in neutropenic children, since their clearance is too high to maintain steady state concentrations at such high levels. Thus, dosing recommendations are based on the 100% *f*T > MIC target. Whereas most other studies consider a PTA of 90% or greater as optimal, a PTA of 95% or greater was considered ideal in this study. Given the stakes of antimicrobial therapy in high‐risk patients with febrile neutropenia, any significant risk of treatment failure is unreasonable, and led us to select a more stringent threshold.

The present study was not designed to assess clinical outcome following continuous piperacillin‐tazobactam infusions, and dosing recommendations rely on the concentration‐time profile. In adults, most studies support improved clinical outcomes and reduced mortality rates with prolonged infusions.^15^, ^16^, ^45^ However, in a multicenter randomized trial, Dulhunty et al^14^ found no difference in outcome between intermittent administration and continuous infusions in adults with severe sepsis. In neutropenic children, clinical outcome has mainly been evaluated in small, unblinded, single‐center trials,^46^, ^47^ and no consistent clinical benefit has been confirmed. While Knoderer et al^47^ found an association between extended infusion and improvements in clinical cure at day 21, Solórzano‐Santos et al^46^ found no significant difference in clinical outcome or mortality between intermittent administration and continuous infusion regimens. Thus, prospective trials evaluating clinical outcomes following continuous infusion in this population are warranted.

Continuous infusion has also proven cost‐effective and safe, with a low risk of dosing errors.^46^, ^48^ Since continuous infusion allows administration of the same dose by one daily procedure and enables homed‐based antimicrobial therapy for clinically stable patients, indwelling catheter‐manipulations, nursing resources and pharmacy costs are reduced. Implementation of prolonged infusions as standard of care has been described as feasible in a pediatric setting.^7^, ^19^, ^49^ In the present study, the patient's clinical condition allowed home‐based antimicrobial therapy in 59 of 68 (87%) fever episodes.

This study has some limitations. First, the study was conducted at a single center, and extrapolation of the results may be limited by variations in pathogen distribution and antimicrobial susceptibility. Still, we present a robust PK model built on 674 samples collected in 78 children (aged 1–17 years) with various malignancies. Previous piperacillin PK studies in neutropenic children included fewer samples^7^, ^8^, ^34^ (23–62 samples) or concentrated on specific age groups.^8^ Although the use of an institutional MIC distribution may limit the transferability of our results to other sites, we argue, that this is compensated by a concomitant evaluation of the EUCAST^26^ susceptibility breakpoint MIC for *P. aeruginosa*. Unfortunately, premature cessation of antimicrobial therapy, change of antimicrobial agent and patients receiving homed‐based antimicrobial therapy, prevented obtainment of all three blood samples in some patients. Although this would have strengthened the PK data, addition of intermittent administration data enhanced the robustness of the final PK model and allowed *f*T > MIC and PTA to be to quantified more precisely. Samples were collected in blood only, which may represent an imprecise surrogate of drug concentrations at the infective site due to a highly variable tissue penetration in severely ill patients. This method implies a risk of overestimating tissue concentrations, particularly in case of deep‐seated infections, such as intra‐abdominal infections and pneumonia.^11^ To allow a further refinement of the dosing regimen for the youngest children, this age group should ideally contain more individuals. Besides, the mix of underlying malignancies precluded assessment of possible PK variations between different types of pediatric malignancies. Finally, as optimal tazobactam PK/PD targets have not been identified in neutropenic children, the PK model was built on the piperacillin component only. While tazobactam has limited antimicrobial activity itself, sufficient 𝛽‐lactamase‐inhibitor concentrations are required to ensure optimal effect of piperacillin against 𝛽‐lactamase‐producing bacteria.^19^

In conclusion, this prospective PK study of children with cancer receiving continuous piperacillin‐tazobactam infusion confirmed, that the optimal dosing regimen to reach the *P. aeruginosa* breakpoint (16 mg/L) for a 100% *f*T > MIC target, was continuous infusion at a dose of 300 mg/kg/day. The strict target of 50/100% *f*T > 4xMIC (64 mg/L), was not attained by any dosing regimen at recommended doses in this population. Thus, based on piperacillin exposure, continuous infusion is supported for optimal PTA against the least susceptible strains infecting children with cancer. Prospective trials evaluating safety and the association between continuous infusion and clinical outcome in this population are warranted.

## CONFLICT OF INTEREST

Birgitte K. Albertsen is sponsor for the investigator initiated NOR‐GRASPALL 2016 study. Speaker and/or Advisory Board Honoraria from Erytech (2020) and Servier (2021). For the remaining authors, no conflicts of interest were declared.

## AUTHOR CONTRIBUTIONS

*Conceptualization*, S.F.M., H.S.; *Methodology*, A.T., L.E.F., E.I.N., S.F.M., M.W., H.S.; *Software*, A.T., L.E.F., E.I.N.; *Validation*, A.T., L.E.F., S.F.M.; *Investigation*, S.F.M., H.S.; *Formal Analysis*, A.T., L.E.F., E.I.N., S.F.M.; *Resources*, H.S., B.K.A. via Danish Childhood Cancer Foundation and Holm's Memorial Trust; *Data Curation*, A.T., S.F.M.; *Writing—Original Draft*, S.F.M.; *Writing—Review & Editing*, S.F.M., A.T., L.E.F., E.I.N., M.W., H.S., B.K.A.; *Visualization*, A.T., S.F.M.; *Supervision*, H.S., B.K.A.; *Project Administration*, S.F.M.; *Funding Acquisition*, S.F.M., H.S., B.K.A.

All authors had full access to the data in the study and take responsibility for the integrity of the data and the accuracy of the data analysis.

## ETHICAL STATEMENT

The study was conducted in accordance with the ethical and registrational standards described in the Declaration of Helsinki and the European Medicines Agency Guidelines for Good Clinical Practice, and was monitored by the Good Clinical Practice‐unit at Aarhus University, Denmark. The study was approved by the Central Denmark Region Committee on Health Research Ethics (registration no. 1‐10‐72‐427‐17), the Danish Medicines Agency (Clinicaltrialsregister.eu registration no. 2017‐004281‐10) and the Danish Data Protection Agency (registration no. 1‐16‐02‐924‐17). Written informed consent was obtained from both parents or legal guardians. In accordance with Danish law, children aged 15–17 years were allowed to provide written informed consent for themselves, in close collaboration with their parents or legal guardians.

## Supporting information


**FIGURE S1** Final model fit diagnostics. The figure shows visual predictive check based on the final model for the merged data set (A) and stratified by study (B). The blue dashed lines and shaded areas representing the median, 5th and 95th percentiles of model simulations and their corresponding 95% confidence intervals, while the red solid lines represent the median, 5th and 95th percentiles of the observed data. IA, intermittent administration; CI, continuous infusion.

**FIGURE S2** Piperacillin doses required to achieve steady state concentrations of 64 mg/L, according to body weight. To reach a piperacillin steady state concentration of 64 mg/L in 95% of the 10 000 children, required doses for children with different body weights are represented by the dashed vertical lines: 1018 (15 kg), 796 (40 kg) and 705 (65 kg) mg/kg/day.

## Data Availability

The data that support the findings of this study are available on request from the corresponding author. The data are not publicly available due to privacy or ethical restrictions.
