# Supplementary figures and images for: Continuous infusion of piperacillin‐tazobactam significantly improves target attainment in children with cancer and fever
Source: Cancer Rep (Hoboken). 2021 Nov 18;5(10):e1585. doi: 10.1002/cnr2.1585 (PMC9575485; doi:10.1002/cnr2.1585)

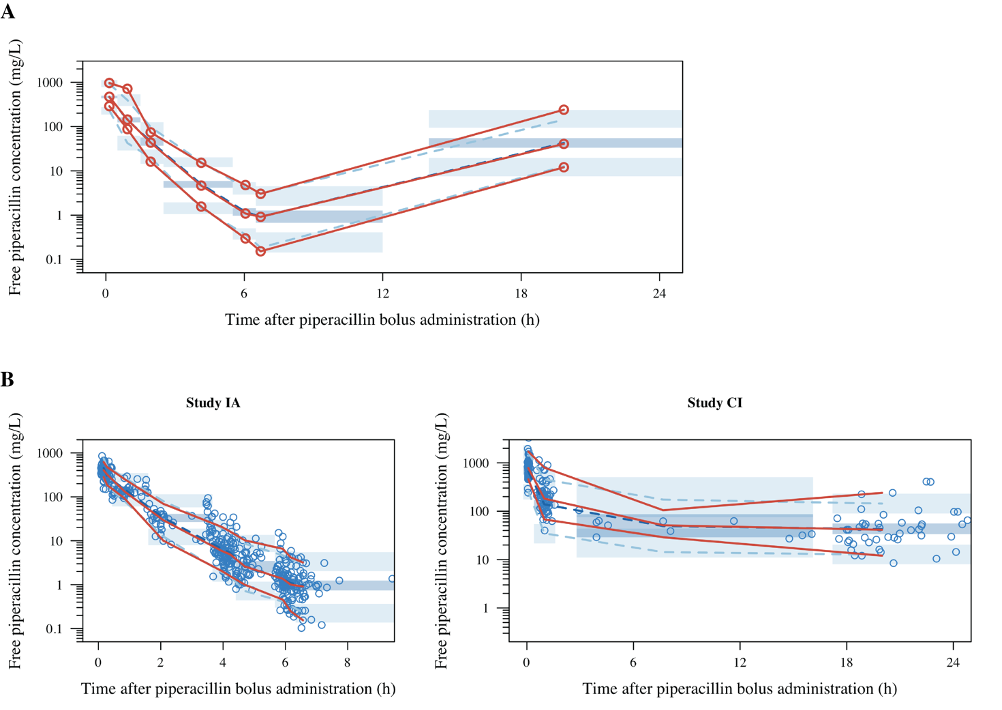

Supplement: Supplementary file 1 — FIGURE S1 Final model fit diagnostics. The figure shows visual predictive check based on the final model for the merged data set (A) and stratified by study (B). The blue dashed lines and shaded areas representing the median, 5th and 95th percentiles of model simulations and their corresponding 95% confidence intervals, while the red solid lines represent the median, 5th and 95th percentiles of the observed data. IA, intermittent administration; CI, continuous infusion. [file CNR2-5-e1585-s002.png]

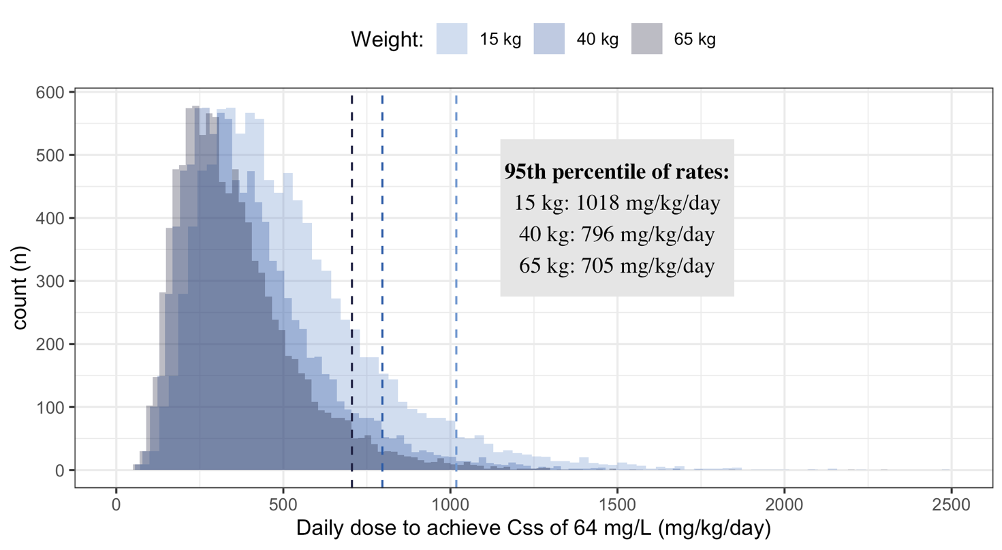

Supplement: Supplementary file 2 — FIGURE S2 Piperacillin doses required to achieve steady state concentrations of 64 mg/L, according to body weight. To reach a piperacillin steady state concentration of 64 mg/L in 95% of the 10 000 children, required doses for children with different body weights are represented by the dashed vertical lines: 1018 (15 kg), 796 (40 kg) and 705 (65 kg) mg/kg/day. [file CNR2-5-e1585-s001.png]
